# Genomic evidence of lung carcinogenesis associated with coal smoke in Xuanwei area, China

**DOI:** 10.1093/nsr/nwab152

**Published:** 2021-08-16

**Authors:** Honglei Zhang, Chao Liu, Li Li, Xu Feng, Qing Wang, Jihua Li, Shaobin Xu, Shuting Wang, Qianlu Yang, Zhenghai Shen, Jinhua Su, Xiaosan Su, Ruifen Sun, Xuhong Zhou, Junliang Wang, Yongchun Zhou, Baowei Jiao, Wanbao Ding, Xianbao Cao, Yue Wang, Yunchao Huang, Lianhua Ye

**Affiliations:** Center for Scientific Research, Yunnan University of Chinese Medicine, China; Department of Nuclear Medicine, Third Affiliated Hospital of Kunming Medical University, Yunnan Cancer Hospital, China; Biotherapy Center, Third Affiliated Hospital of Kunming Medical University, Yunnan Cancer Hospital, China; Center for Scientific Research, Yunnan University of Chinese Medicine, China; Department of Oncology, Qujing First People's Hospital, China; Qujing Center for Disease Control and Prevention, China; Supercomputing Platform of Kunming Institute of Zoology, Kunming Biological Diversity Center of Instruments, Kunming Institute of Zoology, Chinese Academy of Sciences, China; Department of Thoracic Surgery II, Third Affiliated Hospital of Kunming Medical University, Yunnan Cancer Hospital, China; Department of Thoracic Surgery I, Third Affiliated Hospital of Kunming Medical University, Yunnan Cancer Hospital, China; Department of Thoracic Surgery I, Third Affiliated Hospital of Kunming Medical University, Yunnan Cancer Hospital, China; Department of Thoracic Surgery, Xuanwei People's Hospital, China; Center for Scientific Research, Yunnan University of Chinese Medicine, China; Center for Scientific Research, Yunnan University of Chinese Medicine, China; Center for Scientific Research, Yunnan University of Chinese Medicine, China; Center for Scientific Research, Yunnan University of Chinese Medicine, China; Department of Thoracic Surgery I, Third Affiliated Hospital of Kunming Medical University, Yunnan Cancer Hospital, China; State Key Laboratory of Genetic Resources and Evolution, Kunming Institute of Zoology, Chinese Academy of Sciences, China; Center for Excellence in Animal Evolution and Genetics, Chinese Academy of Sciences, China; KIZ-CUHK Joint Laboratory of Bioresources and Molecular Research in Common Diseases, Kunming Institute of Zoology, Chinese Academy of Sciences, China; Department of Oncology, Yan’an Affiliated Hospital of Kunming Medical University, China; Department of Otolaryngology, First People's Hospital of Yunnan Province, the Affiliated Hospital of Kunming University of Science and Technology, China; The Affiliated Hospital of Guizhou Medical University, China; Department of Thoracic Surgery I, Third Affiliated Hospital of Kunming Medical University, Yunnan Cancer Hospital, China; Department of Thoracic Surgery I, Third Affiliated Hospital of Kunming Medical University, Yunnan Cancer Hospital, China

Xuanwei area, in southwestern China, harbors the highest female lung cancer rate in the country (Supplementary Table 1) [1]. Epidemiological studies have shown that lung cancer incidence in Xuanwei area is associated with the use of different types of local coal for household cooking and heating [2]. The genomic landscape of Xuanwei female adenocarcinoma (XWFA), the most distinctive feature of lung cancer in Xuanwei, has yet to be elucidated systematically. Here, we provide the fundamental resource of genomic datasets for further exploration of XWFA molecular mechanisms and development of targetable therapy.

The patients recruited for this study were 117 non-smoker females with untreated primary lung adenocarcinoma (LUAD) from the Xuanwei area, who were receiving surgical treatment at Yunnan Cancer Hospital (Supplementary Table 2). Samples were taken for whole-exome sequencing (WES) (112 pairs of tumor-normal), and 33 normal and 115 tumor samples were sequenced with mRNA-Seq technology. Datasets of 168 TCGA-LUAD female smokers (TLSF) and 102 TCGA-LUAD female non-smokers (TLNF) were adopted from The Cancer Genome Atlas (TCGA) program for genomic comparison between lung cancer associated with cigarettes and that associated with smoky coal [3] (Supplementary Table 3). There were no significant differences in the distribution of samples from different pathologic stages among the XWFA, TLSF and TLNF cohorts (Fig. 1a).

**Figure 1.**
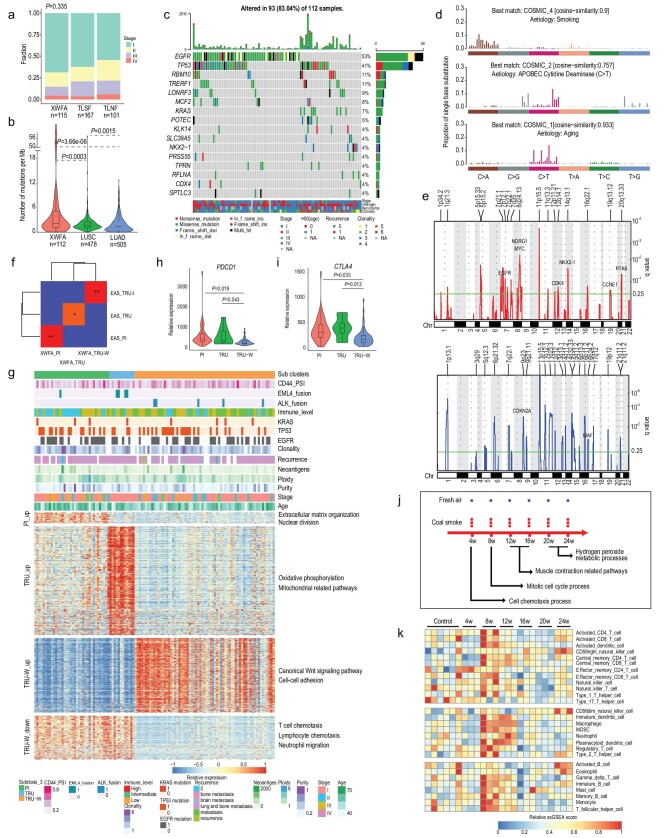
Molecular characterization of XWFA and rat model recapitulation of lung cancer initiation. (a) Comparison of distributions of samples from different pathology stages (I, II, III and IV) across the XWFA, TLSF and TLNF cohorts. Chi-square test was used to calculate *P* value. (b) Violin plot showing the tumor mutation burden (TMB) across the XWFA, TCGA-LUAD and TCGA-LUSC cohorts. The box indicates the interquartile range (IQR), the middle line indicates median, whiskers indicate the highest and lowest values within 1.5× IQR away from the box, and dots plot values >1.5× IQR away from the box. (c) Co-mutation plot of tumor samples from the XWFA cohort. Significantly mutated genes were identified with MutSig2CV algorithm (FDR corrected *P* < 0.25) and oncodriveCLUST (FDR corrected *P* < 0.1) and were ranked in order of decreasing prevalence. Clinical features such as pathology stages, ages, recurrence status and clonality were indicated. (d) Top three mutation signatures derived from single nucleotide variants were identified with Cosmic Mutational Signatures (version 2.0). (e) Focal-level CNV across chromosomes 1–22 in XWFA cohort, with GISTIC FDR q values on the y axis. Amplifications were labeled red (top) and deletions were labeled blue (bottom). Selected genes in the Cancer Gene Census (CGC) were labeled in the significant peak regions. (f) Association analysis of subgroups identified in the XWFA and EAS cohorts with Submap. Significant correspondence between subgroups highlighted in red with Bonferroni adjusted *P* values. ^*^: FDR < 0.1; ^*^^*^: FDR < 0.01. (g) Phenotypes of RNA-based subgroups in XWFA. The annotation rows showing the genomic and clinicopathologic features for each patient. The heat map showing the normalized mean expression of subgroup differentially expressed genes. Representative GO biological processes were labeled right. (h, i) Comparison of *PDCD1* (h) and *CTLA4* (i) expression among PI, TRU and TRU-W clusters in XWFA cohort. (j) Schematic showing the overall experimental design for the rat_coal model (top). Red dot: rats treated with smoke from local smoky coal; blue dots: rats treated with fresh air. The arrow below the timeline indicates the most enriched disturbed biological processes during smoke treatment. (k) Heatmap showing the relative infiltration of immune cells derived from RNA-based ssGSEA scores in lung tissue across six time points. Two-sided Mann-Whitney U test was used for *P* value calculation in (b, h and i).

In the XWFA cohort, 35 729 somatic mutations comprising 34 287 single-nucleotide variants (SNVs) and 1442 insertions or deletions (INDELs) were identified (Supplementary Methods). Compared with the TCGA lung cancer samples, XWFA samples possessed higher mutation burdens (median = 2.11) than lung squamous cell carcinomas (LUSC) (median = 1.63) and LUAD (median = 1.41) (Fig. 1b). This suggests substantial differences in the mutational genomic landscape between the XWFA and Western cohorts. Furthermore, MutSig2CV (for details of software, refer to the Supplementary Methods) and oncodriveCLUST algorithms were adopted jointly to identify significantly mutated genes (SMGs) in the XWFA cohort. As demonstrated in Fig. 1c, the most prominent cancer-related variations observed in the XWFA cohort were *EGFR* mutations (found in 52.68% of tumors), followed by mutations in *TP53* (41.07%), *RBM10* (10.71%), *KRAS* (7.14%) and *NKX2-1* (4.46%). Four genes including *EGFR*, *KRAS*, *TPRN* and *SPTLC1*, were identified as driver genes using the oncodriveCLUST algorithm. As SMGs often serve as gatekeepers, which may be targetable or serve as predictive biomarkers for immune checkpoint therapy [4], the relationships among SMGs, mutation load and neoantigens derived from mutations were next examined (Supplementary Fig. 1). The mutation load and the number of neoantigens were significantly correlated (Supplementary Fig. 1a). Higher mutation loads and more neoantigens were observed in *TP53*, *KRAS*, *RBM10* and *POTEC* mutant samples than in the respective WT samples (Supplementary Fig. 1d–k). Taken together, the canonical and novel SMGs identified in the XWFA cohort, which were suspected to be targetable or explored as biomarkers, merit further investigation. LUAD-specific driver gene lists were collected and are listed in Supplementary Table 4.

Mutational signatures were further investigated to infer the mutational process during XWFA initiation. Mutation spectrum results showed that the substitution pattern and transversion/transition ratio of the XWFA cohort were similar to those of the TLSF cohort and both showed high C > A substitution (Supplementary Fig. 2). Three mutation signatures including ‘smoking’ (COSMIC_4), ‘APOBEC’ (COSMIC_2) and ‘Aging’ (COSMIC_1) were identified using the nonnegative matrix factorization (NMF) algorithm in the XWFA cohort (Fig. 1d). Polycyclic Aromatic Hydrocarbons (PAHs) and nicotine-derived nitrosamines, two smoking carcinogens reported to be strongly associated with C > A transversion hotspots [5], were also found in high levels in local smoky coal from Xuanwei [2]. This explained the similarities in mutation spectra and mutation signatures between the XWFA and TLSF cohorts, which further supports the hypothesis that the high lung cancer rate in Xuanwei area results, at least partially, from domestic use of local smoky coal.

Copy number variations (CNVs) play pivotal roles in tumor initiation. We identified significantly altered CNVs with Sequenza and the Genome Identification of Significant Targets in Cancer (GISTIC) 2.0 algorithm in the XWFA cohort (Fig. 1e, Supplementary Fig. 3 and Supplementary Table 5). Generally, numbers of CNV-affected genes (both amplification and deletion) in the XWFA cohort were higher than in the TLNF and TLSF cohorts (Supplementary Fig. 3e and 3f). However, many focal amplification CNVs around driver genes such as *MYC*, *PVT1* and EGFR, and deletion CNVs such as *CDKN2A* and *CDKN2B*, were identified in all three cohorts (Fig. 1e, Supplementary Fig. 3a–f), which suggests pivotal roles for those genes in initiation of lung cancer. Both amplification and deletion genes detected by ABSOLUTE and Sequenza in the XWFA cohort were comparable, with only a small proportion of genes identified software-specifically (Supplementary Fig. 3g), indicating stable detection of the CNVs. Overall, our results suggest that the XWFA cohort had more genomic CNVs than the TLSF and TLNF cohorts, which further suggests substantial differences in the genomic landscape between XWFA and Western cohorts and that the significantly CNV-affected genes need further investigation.

Unsupervised clustering of RNA-seq data from XWFA tumor samples revealed three subgroups and the SubMap module was applied to compare the subgroups between the XWFA (N = 115) and ESA [6] (N = 230) cohorts. Although we found subgroups from the XWFA cohort that were significantly correlated to the PI, TRU and TRU-I subgroups from the ESA cohort (Fig. 1f), we also found a subgroup highly expressing Wnt signaling pathway genes and designated this the TRU-W subgroup, corresponding to the TRU-I subgroup in the ESA cohort. To further explore the TRU-W subgroup, we identified its up- and down-regulated genes and found that low immune infiltration was the most remarkable feature (Fig. 1g). This result indicates that WNT/β-catenin pathway activation correlates with immune exclusion across human cancers. Furthermore, we found that expression of *PDCD1* and *CTLA4* were significantly lower in the TRU-W subgroup compared with PI and TRU samples (Fig. 1h and 1i). All these results suggest that the TRU-W subgroup from the XWFA cohort formed a specific cluster with low immune infiltration and high Wnt signaling, which should be considered in further immunotherapy. Clinical and molecular features (including immune cell infiltration status, Supplementary Fig. 8 and Supplementary Methods) among the subgroups in the XWFA cohort were further compared. The TRU-W subgroup was enriched with EML4 fusion events and low and intermediate immune infiltration samples (Supplementary Fig. 5k and 5q); other features showed no significant differences (Supplementary Fig. 5a–j and m–p).

To explore experimentally the role of emissions from indoor combustion of C1 bituminous coal in the initiation stage of XWFA, a lung cancer model (rat_coal model) derived from female F344 rat was established (Supplementary Methods, Supplementary Fig. 6a, Fig. 1j and Supplementary Fig. 7). We firstly investigated the biological process alterations in both rat_coal and mouse_cigarette models [7]. Our results showed clear step-wise alterations of biological processes in both models during smoke treatment. Cell chemotaxis processes, mitotic cell cycle process, muscle contraction-related pathways and hydrogen peroxide metabolic processes were mostly altered after 4 weeks, 8 weeks, 12–16 weeks, 20–24 weeks treatment in the rat_coal model (Fig. 1j and Supplementary Fig. 8). Parallelly, immune response, regulation of cell cycle, immune response, smooth muscle construction and oxidation-related process after 1 week, 1 month, 3 months, 6 months and 9 months treatment were identified in the mouse_cigarette model (Supplementary Fig. 6b and Supplementary Fig. 9). These parallel step-wise alterations of biological process from both the rat_coal model and mouse_cigarette models reflected progressive tumor initiation starting from inflammation.

We further investigated the trend of tumor-infiltrating lymphocytes (TILs) during tumor initiation with a single-sample gene set enrichment analysis (ssGSEA) method based on RNA-Seq profiling. The remarkable feature was the wave of TIL profiles in both models. Specifically, TILs rose notably after 8 weeks treatment, decreased gradually to 20 weeks and increased again at 24 weeks in the rat_coal model (Fig. 1k). A parallel trend was observed in the mouse_cigarette model (Supplementary Fig. 6c). This trend of TILs was correlated with alteration of biological pathways in lung tissues. Specifically, activated CD4/CD8 T cells, CD56bright/dim natural killer cells and activated B cells were more enriched at 24 weeks, which was accompanied by up-regulation of hydrogen peroxide metabolic processes. It has been proved that hydrogen peroxide-induced oxidative stress further triggered an innate immune response in A549 cells [8]. These results reflect the cross-talk between tissue cells and TILs during tumor initiation. Further exploration of expression of immune-related genes revealed several potential therapeutic targets in lung cancer initiation (Supplementary Fig. 6d). For example, B and T lymphocyte attenuator (BTLA), belonging to the CD28 superfamily and similar to programmed cell death-1 (PD-1) and cytotoxic T lymphocyte associated antigen-4 (CTLA-4) in terms of its structure and function, induces immunosuppression by inhibiting B and T cell activation and proliferation [9]. Another promising target is KDR (VEGFR-2), which is the main mediator of VEGF-induced tumor cell proliferation, migration, survival and increased permeability [10]. All the above mouse homologous genes showed remarkable up-regulation after 24 weeks in the rat_coal model (Supplementary Fig. 6d), indicating a suppressive state of both adaptive immune and innate immunity by these suppressors, which also served as promising therapeutic targets during the initiation stage of lung cancer.

Taking all the results together, our study establishes a valuable resource for XWFA, provides insight into the initiation process and indicates that therapies targeting the SMGs, early-stage pathway alterations or blocking immune-cancer cross-talk, show potential and merit further investigation.

## DATA AVAILABILITY

Clinical data (deidentified) are listed in Supplementary Table 2. Raw sequencing data including WES and RNA-seq datasets from human samples have been deposited in the Genome Sequence Archive under the accession code HRA000124. The RNA-seq dataset from rat model samples has been deposited in the Gene Expression Omnibus (GEO, https://www.ncbi.nlm.nih.gov/geo/) under the accession code GSE162001 [https://www.ncbi.nlm.nih.gov/geo/query/acc.cgi?acc=GSE162001]. RNA-seq dataset for mouse_cigarette model analyses were downloaded from GEO accession: GSE76205 [https://www.ncbi.nlm.nih.gov/geo/query/acc.cgi?acc=GSE76205]. RNA-seq and somatic mutation datasets of TCGA-LUAD and TCGA-LUSC were downloaded from XENA (https://xena.ucsc.edu/). All other relevant data are available within the article, Supplementary data or available from the authors upon request.

## Supplementary Material

nwab152_Supplemental_Files
