# Supplementary material for: Genomic evidence of lung carcinogenesis associated with coal smoke in Xuanwei area, China
Source: Natl Sci Rev. 2021 Aug 16;8(12):nwab152. doi: 10.1093/nsr/nwab152 (PMC8692936; doi:10.1093/nsr/nwab152)
Supplement: nwab152_Supplemental_Files [file nwab152_supplemental_files.zip › Supplementary_Information.docx]

# SUPPLEMENTARY INFORMATION

**Genomic evidence of lung carcinogenesis associated with coal smoke in Xuanwei area, China**

Zhang et al.

***Corresponding authors (Emails):**

[Lhye1204@aliyun.com](mailto:Lhye1204@aliyun.com)

[huangych2001@aliyun.com](mailto:huangych2001@aliyun.com)

[hlzhang2014@163.com](mailto:hlzhang2014@163.com)

**This PDF document contains:**

Supplementary Methods

Supplementary Figures S1-S9

**Supplementary Methods:**

**Software mentioned in the main text**

- MutSig2CV^1^
- oncodriveCLUST algorithms^2^
- nonnegative matrix factorization (NMF) algorithm (R package v.0.22)^3,4^
- Sequenza(v3.0.0)^5^
- Genome Identification of Significant Targets in Cancer (GISTIC, version 2.0.22) ^6^
- ABSOLUTE^7^
- SubMap^8^
- single-sample gene set enrichment analysis (ssGSEA, R package v10.0.2) ^9^

**Patients and sample information**

A total of 117 female patients from the Xuanwei area with previously untreated lung adenocarcinoma were retrospectively enrolled according to the following defined criteria: female patients who 1) were diagnosed with previously untreated lung adenocarcinoma; 2) came from the Xuanwei area; 3) had smoked less than 100 cigarettes in their lifetime; 4) exhibited no evidence of distant metastasis at diagnosis; and 5) had been subjected to pathological assessment (exclusion criteria with necrosis levels > 20%). Sample collection was conducted between May 2016 and July 2019 in Yunnan Cancer Hospital. The study was approved by the ethical committees of Yunnan Cancer Hospital & The Third Hospital of Kunming Medical University and all patients provided written informed consent to participate in genomic studies.

Follow-up within this cohort of patients was completed on November 2019, and the median follow-up was conducted at 11 months. Fourteen patients experienced relapse and/or metastatic progression at the time of the last follow-up. Relapse-free survival (RFS) was defined as the time from diagnosis to first recurrence. Surgically resected tumours and adjacent normal tissue were collected, kept in RNAlater (Thermo Fisher Scientific, USA) overnight and stored at -80°C for long-term preservation. Majority of the tumours analysed were stage I or II (n=93); the remainder were stage III or IV (n=17).

DNA and RNA fractions were isolated from the tissue using an AllPrep DNA/RNA mini kit (Qiagen). Frozen tissue was homogenized with tissue disruptor. DNA was selectively collected on a spin column and the column was then washed. DNA was eluted in 0.1 x TE buffer. TRIzol was added to the flow-through, which contained RNA, and the solution heated at 65°C for 5 minutes. Chloroform was added and the phases were separated via centrifugation. Ethanol was added to the aqueous phase to provide appropriate binding conditions for RNA. The sample was then applied to a RNeasy spin column, treated with DNase I to remove residual contaminating DNA, then washed and eluted in 0.1 x TE buffer.

**Whole-exome sequencing and somatic mutation calling**

Whole-exome sequencing (WES) applied to tumour and matched normal DNA was performed on an Illumina HiSeq 2000 using the 2 × 150-bp paired-end sequencing method. Agilent SureSelect All Exon V2 was used to capture the exon regions. The average sequence coverage of normal samples and tumour samples was 118.8 (SD=24.3) and 122.3 (SD=25.0), respectively. Clean FASTQ files were obtained by filtering the following three types of reads: 1) reads with adapter sequences; 2) reads with the number of N bases exceeding 10% of the total number of read bases; and 3) reads with low-quality bases (<5) exceeding 50% of the read length. Clean reads were then aligned to the GRCh37 reference genome using bwa (v0.7.10) with the mem algorithm^10^. Then, the resulting SAM files were converted to the BAM format using samtools (v0.1.19)^11^ and subsequently de-duplicated and sorted using Picard (v2.20.2). Base quality recalibration and INDEL realignment were performed using GATK (v4.1.2)^12^. Reads with low mapping quality (< 30) were further filtered with samtools (v0.1.19). Mutect2 ^13^ was performed to call somatic SNPs and INDELs.

To further filter the raw vcf files, GetPileupSummaries was used to calculate pileup statistics for inferring contamination. The gnomAD resource file af-only-gnomad.hg37.vcf.gz was set as the site VCF file, which summarized the alt and ref counts along with population allele frequency^14^. FilterMutectCalls from GATK were used to filter VCF files with contamination adjustment.

**Mutation annotation**

SNV and short INDEL calls generated from mutect2 results were merged and then annotated using the UCSC hg19 database with annovar^15^. ExaC (r0.2), 1000 Genomes (phase 1), Mills, COSMIC (v74), and dbSNP 138 were used for the variant annotation.

**Mutation load comparison among XWFA, TCGA-LUAD and TCGA-LUSC cohorts**

Tumor mutation burden (TMB) was represented by the number of mutations per Mb. Specifically, somatic mutation files including LUAD_mc3.txt and LUSC_mc3.txt from Multi-Center Mutation Calling in Multiple Cancers (MC3) project, which has developed pipelines to uniformly apply many mature tools across the TCGA sequencing project, were downloaded from UCSC Xena (http://xena.ucsc.edu/). Target region bed files including gencode.v19.basic.exome.bed for TCGA and agilent_region_exome_v6.bed for XWFA derived from sequencing company (Novogene Co., Ltd.) were used to determine the target size of exon sequencing (192.346066M for TCGA and 60.700153M for XWFA). Mann-Whitney U test was used to calculate *P* values and median values were labeled. The box indicates the interquartile range (IQR), the middle line indicates median, whiskers indicate the highest and lowest values within 1.5× IQR away from the box, and dots plot values >1.5× IQR away from the box.

**Characterization of neoantigens**

NeoPredPipe (Neoantigen Prediction Pipeline) ^16^ was used to predict putative neoantigens and their corresponding recognition potentials. Specifically, NeoPredPipe employed ANNOVAR^15^ to annotate and predict the mutated amino acid sequences from nonsynonymous variants and extract the peptide sequences surrounding the amino acids. Then, NeoPredPipe used the peptide sequences and HLA haplotype results determined by POLYSOLVER^17^ to execute netMHCpan^18^ for the neoantigen predictions.

**Detection of cancer driver genes based on positional clustering**

We used the oncodrive function of maftools^4^ to identify cancer genes (drivers) from a MAF based on the oncodriveCLUST algorithm^2^. The concept is based on the fact that most of the variants in driver genes are enriched at few specific loci (also known as hot spots). This method takes advantage of such positions to identify cancer genes.

**Mutational signature identification**

To identify mutation signatures across XWFA, TLSF and TLNF cohorts, following steps were taken: 1) Obtain the mutation matrix from mutation annotation format (MAF) file; 2) Cophenetic correlations were calculated and elblow plots were drown which help to decide optimal number of signatures. Best possible signature is the value at which cophenetic correlation drops significantly; 3) Non-negative matrix factorization ^3^ was used to decompose the mutation matrix into n signatures. N is determined by above step; 4) Signatures extracted above were compared to signatures from COSMIC database^19^, and cosine similarity was calculated to identified the best match; 5) Signatures were assigned to samples according to relative contributions of these signatures. Maftools (v.2.2.10) R package^4^ was used to characterize and visualize the results from the signatures inferred from NMF. The same processes were performed on the TLSF and TLNF cohorts.

**CNVs and clone number identification**

For XWFA, TLSF and TLNF cohorts, Sequenza (3.0.0)^5^ was adopted to estimate the copy number for each patient with default settings and GISTIC (v.2.0.23)^6^ was used to identify significantly amplified and deleted regions in each cohort. The GISTIC parameters were set as follows: -genegistic 1 -smallmem 1 -broad 1 -brlen 0.5 -conf 0.95 -armpeel 1 -savegene 1 -gcm extreme. Clone numbers (clonality) for each sample in XWFA cohort were inferred with pyclone (0.13.1)^20^ which using sequenza results as input. Dataset from XWAF was also processed with ABSOLUTE^7^ to compute copy-number and the same parameters were used for GISTIC pipeline for the subsequent analysis.

**GO pathway analysis**

Enriched GO biological processes were extracted from DAVID^21^ and Metascape ^22^ and FDR < 0.05 was used to determine the significance.

**Expression subtype detection and comparison among cohorts**

Gene expression profiles from 115 XWFA cohort samples were normalized with edgeR ^23^ and the top 3000 variable genes were used for clustering with using NMF (R package v.0.22) ^3^. Optimal rank parameters were determined with 50 runs of random starting points to acquire appropriate clustering solutions. The smallest cluster in rank=4 clustering solution only contained 6 samples and was excluded from further analysis.

SubMap ^8^ module from the online platform GenePattern was used to compare the three clusters in XWFA and EAS LUAD cohorts. Genes used were isolated commonly in both cohorts (N=2245). Significant correspondences were identified with Bonferroni adjusted *P* < 0.1.

**Tumour-infiltrating immune cell analysis**

Signature genes for specific immune cell types were adopted from previous study ^24^. The immune signature score was calculated using the single-sample gene set enrichment analysis (ssGSEA) method implemented by the R package GSVA^9^. RNAseq datasets from rat_coal model and mouse_cigarette model was also processed with GSVA pipeline.

**Establishment of a rat lung cancer model induced with local smoky coal**

The equipment for the rat experiment was used as described in a previous study ^25^ to stimulate the “open fire pond” used in Xuanwei rural areas, which produce high smoke concentrations. The test equipment consists of four parts (Supplementary Fig. 10a). The experimental animals included 24 F344 rats which were purchased from Beijing Weitong Lihua Experimental Animal Technology Co., Ltd. (scxk (Beijing) 2017-0001). The average weight was 80 ±10 g (Mean ± SD), and all the animals were healthy female rats, with an age of 14-21 days. In the experiment, all the coal was taken from C1 bituminous coal of Laibin, Xuanwei. Briefly, 24 rats were allocated in a control group (6 rats) and an experimental group (18 rats). The experimental F344 rats were exposed to C1 bituminous coal combustion products and the control group was treated with fresh air. Lung tissues were extracted from 6 time points (4 w, 8 w, 12 w, 16 w, 20 w and 24 w) and were processed for mRNA-seq sequencing (Fig. 1m). Pathologically, the lung tissue from experimental group at 4 weeks were dark red; after 8 w, 60% of the lung tissue in the experimental group had black plaque and a large number of small nodules; after 24 w treatment, one experimental rat initiate a large mass in the lung tissue (Supplementary Fig. 11). The animal study was approved by the ethical committees of Yunnan Cancer Hospital & The Third Hospital of Kunming Medical University.

**Statistical analyses**

If not specified, two-sided Fisher’s exact testing was used for *P* value calculations between two categorical variables, while two-sided Mann-Whitney U testing was used between two continuous variables. For multiple testing correction, FDR corrections were performed unless specified otherwise. Pearson correlation method was used to determine the relationship of two variables. All the box plots in figures show the median, inter quartile range (25th and 75th percentiles) and 1.5 times the interquartile range. All analyses were performed using the statistical software environment R.

**Supplementary Figures:**


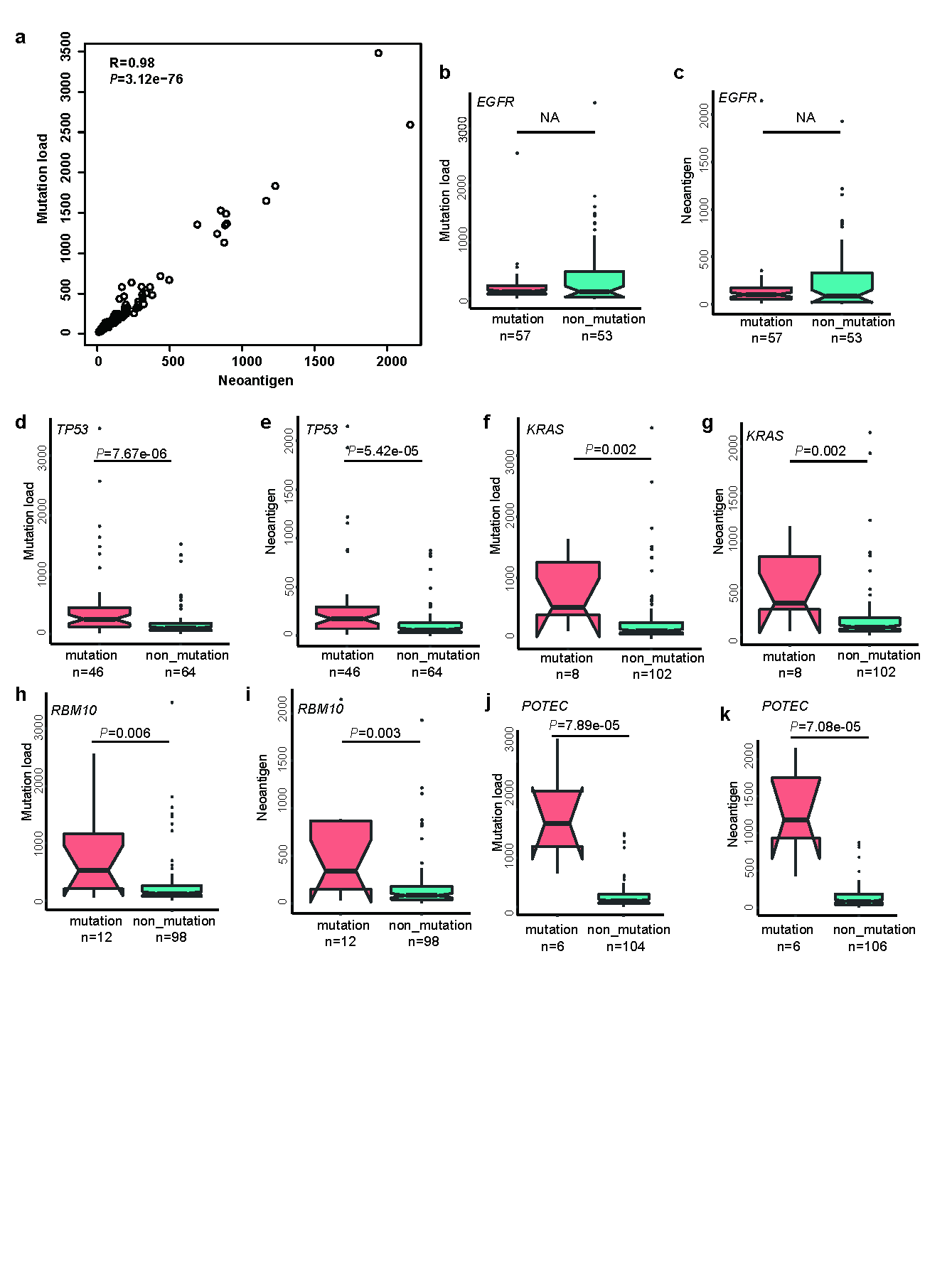
**Supplementary Fig. 1| Associations between driver mutations and mutation load or the number of neoantigens.** (**a**) Correlation of mutation load and the number of neoantigens in XWFA cohort. Pearson correlation coefficient (R) and *P* values were labeled. (**b, d, f, h** and **j**) Association between driver mutation and mutation load. EGFR(**b**), TP53 (**d**), KRAS(**f**), RBM10(**h**), POTEC(**j**). Number of mutational and wide-type samples were shown. (**c, e, g, i** and **k**) Association between driver mutation and the number of neoantigens. EGFR(**c**), TP53(**e**), KRAS(**g**), RBM10(**i**), POTEC(**k**). Number of mutational and non_mutational samples were shown. Mann-Whitney U test was used to calculate *P* values in **b-k** and *P* values were FDR correlated. The boxes for (**b-k**) indicate the interquartile range (IQR), the middle line indicates median, whiskers indicate the highest and lowest values within 1.5× IQR away from the box, and dots plot values >1.5× IQR away from the box.


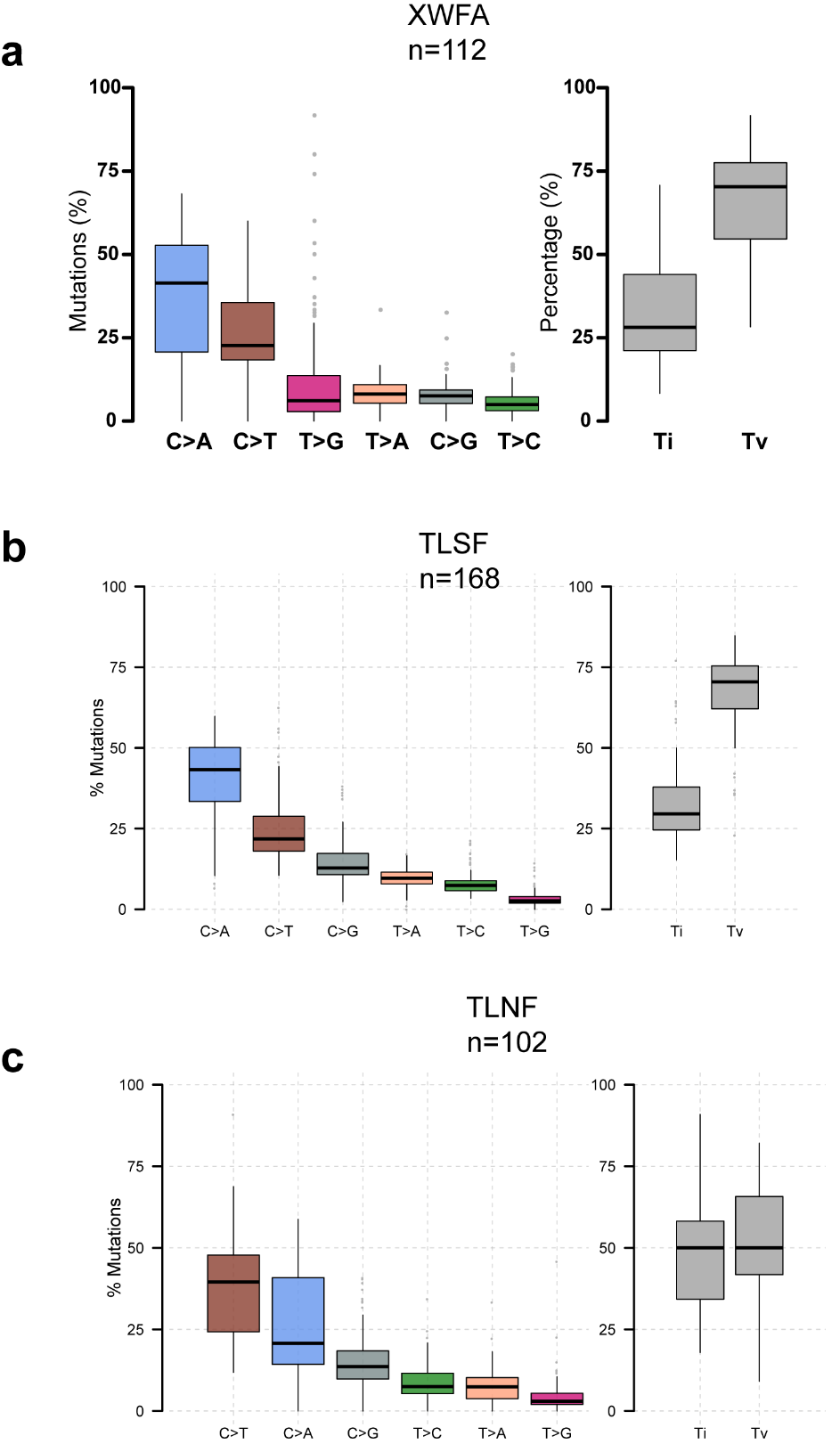


**Supplementary Fig. 2| Mutation spectrum in XWFA, TLSF and TLNF cohorts. (a-c)** Summarized statistics of six different conversions (left) and transitions/transversions (right) based on single-nucleotide polymorphisms (SNPs) identified in the XWFA (**a**), TLSF (**b**) and TLNF (**c**) cohort. The box from (**a-c)** indicates the interquartile range (IQR), the middle line indicates median, whiskers indicate the highest and lowest values within 1.5× IQR away from the box, and dots plot values >1.5× IQR away from the box.


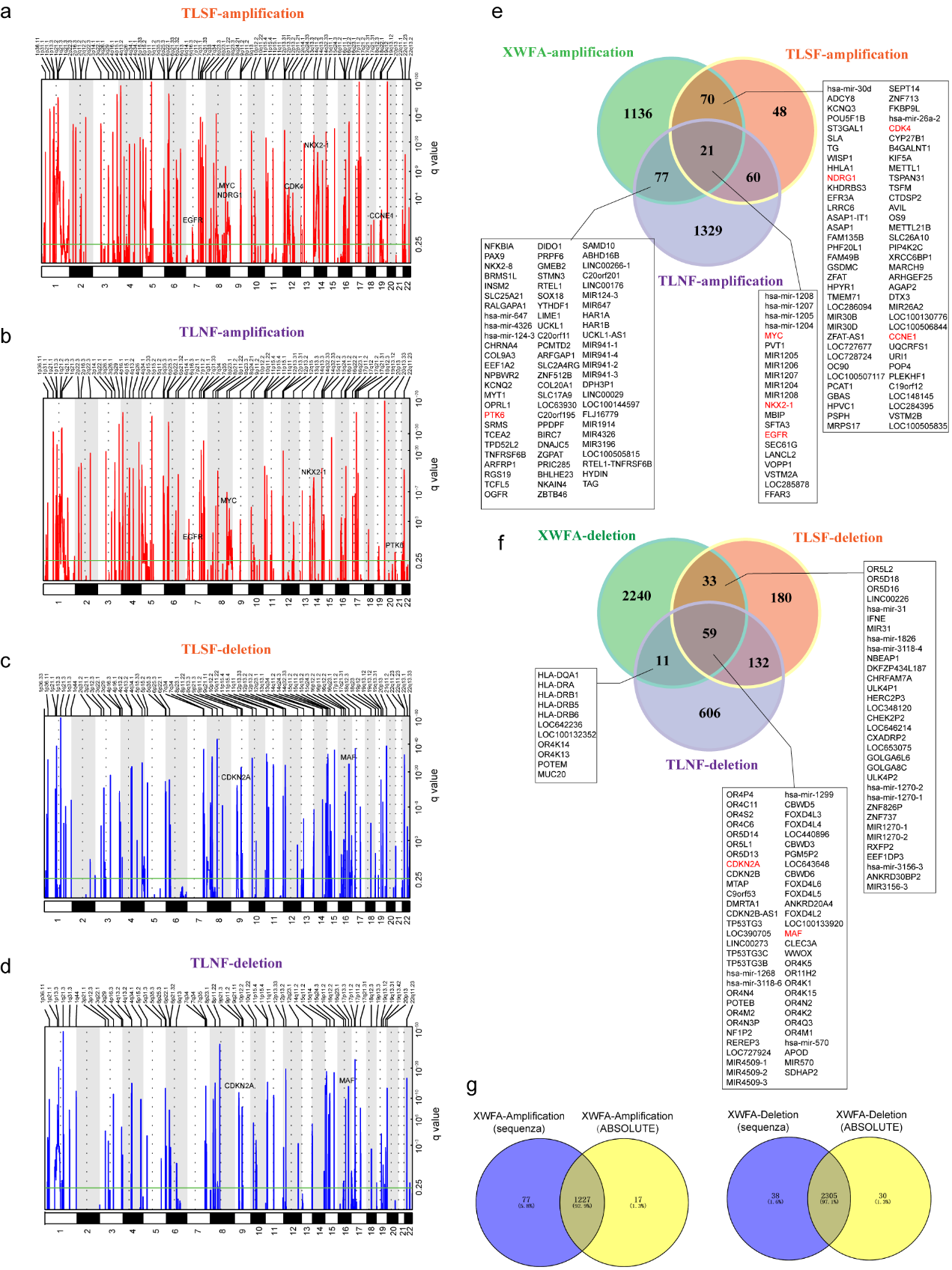


**Supplementary Fig. 3| Focal CNVs analyses in XWFA, TLSF and TLNF.** (**a-d**) Focal-level of CNVs across chromosomes 1–22 in TLSF and TLNF cohort. **a**, TLSF-amplification; **b**, TLNF-amplification; **c**, TLSF-deletion; **d**, TLNF-deletion. GISTIC FDR q values on the right y axis. (**e-f**) Venn plot showing the common amplification (**e**) affected genes or deletion (**f**) genes among XWFA, TLSF and TLNF cohorts. Intersected genes from XWFA cohort were labeled. Cancer Gene Census (CGC) tier 1 genes were marked red. (**g**) Venn plot showing the common and software-specific amplification (left) and deletion(right) genes using sequenza and ABSOLUTE in XWFA cohort.


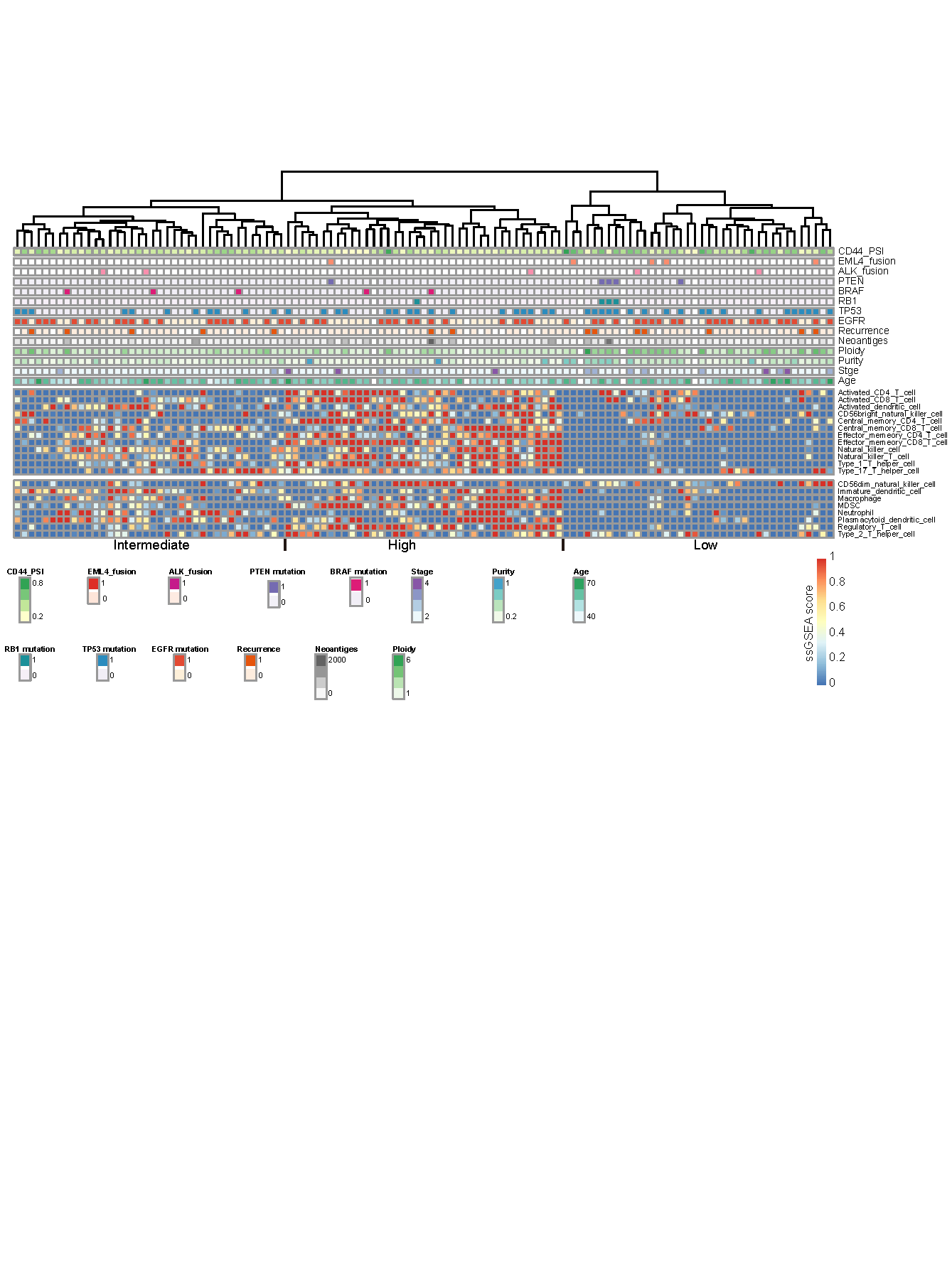


**Supplementary Fig. 4| Enrichment of tumor infiltrating immune cells in XWFA cohort.** Twenty infiltrating lymphocytes were classified into anti-tumour groups and pro-tumour groups according to a previous study ^24^. The mutation status (*EGFR*, *TP53*, *RB1*, *BRAF* and *PTEN*), fusion status (*EML4* and *ALK*) and AS events of CD44:v8 were sample annotated. Clinical information such as patient age, tumour stage, purity, ploidy, number of neoantigens and recurrence status were also shown. Immune infiltration status was defined according the unsupervised clustering and labeled at the bottom of heatmap.


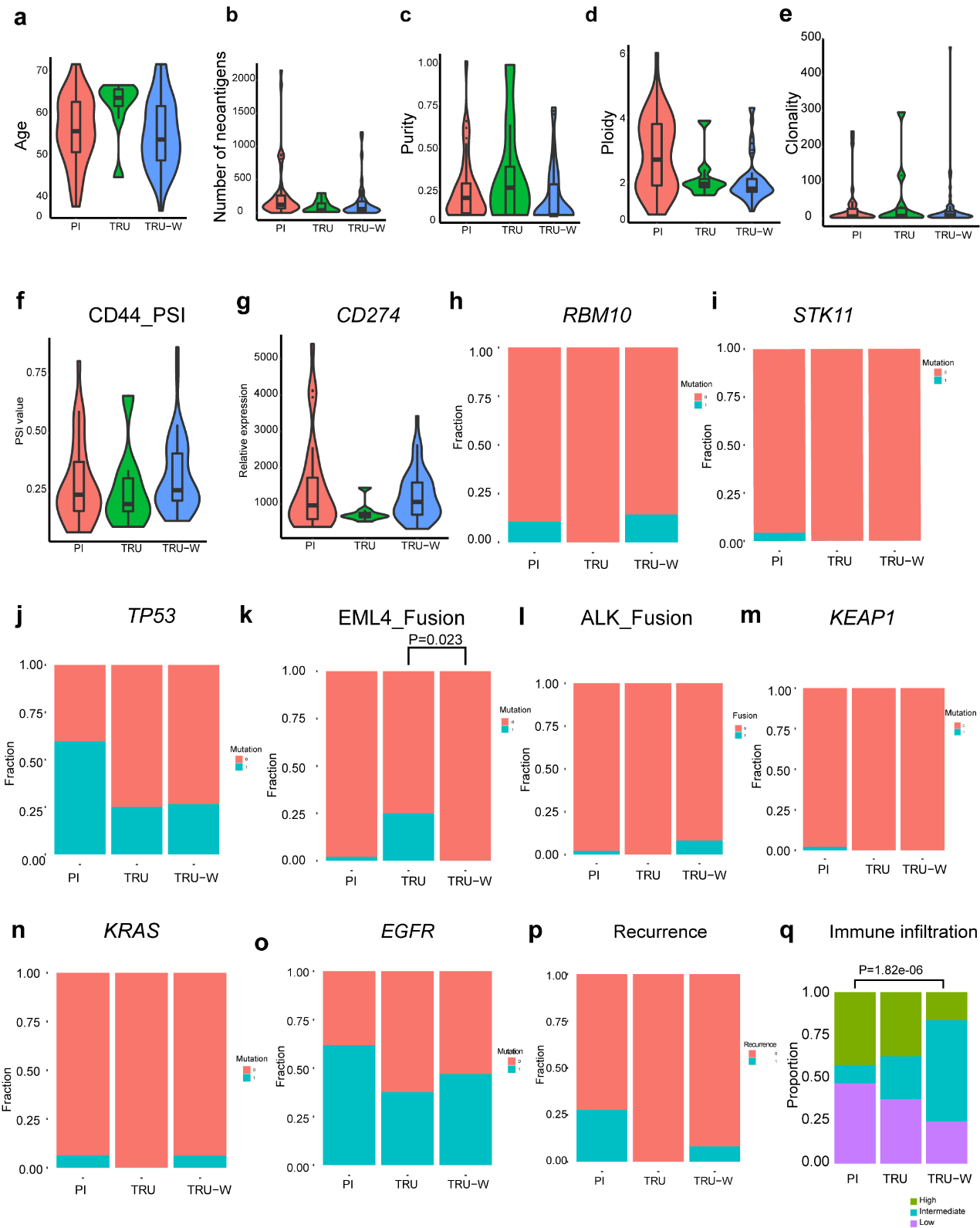


**Supplementary Fig. 5| Comparisons of features among the subgroups in XWFA cohort.** Comparisons of various clinicopathologic (a, Age; b, Neoantigens; c, Purity; d. Ploidy; e, Clonality, p, Recurrence) and genomic features (f, *CD44* v8; g, *CD274* expression; h, *RBM10* mutation; i, *STK11* mutation; j, *TP53* mutation; k, *EML4* fusion; l, *ALK* fusion; m, *KEAP1* mutation; n, KRAS mutation; o, EGFR mutation; q, immune cell infiltration) among PI, TRU and TRU-I clusters in the XWFA cohort. Mann-Whitney U test in **a-g** and Fisher’s exact test in **g-q** was used to test significance. Significant difference was marked in **k**. The boxes from (a-g) indicate the interquartile range (IQR), the middle line indicates median, whiskers indicate the highest and lowest values within 1.5× IQR away from the box, and dots plot values >1.5× IQR away from the box.


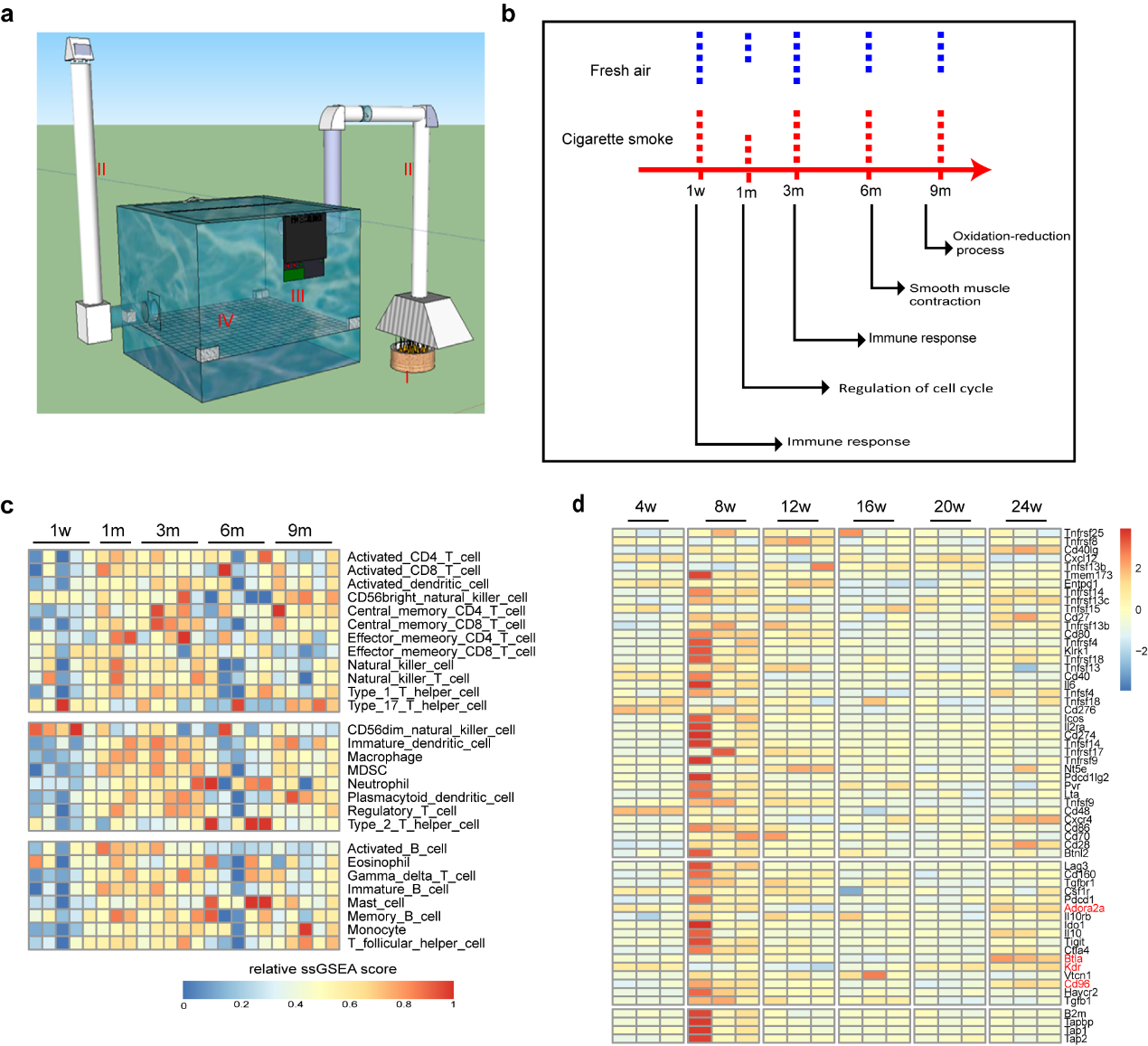


**Supplementary Fig. 6| Establishment and analysis of rat_coal model and mouse_cigarette model.**(**a**) **Equipment used in rat_coal model.** I, coal furnace generator; II, air inlet pipeline; III, sensor device; PM2.5 infrared fine particle sensor; IV, test box: 1 m * 1 m * 1 m glass box. (**b**) Schematic showing the overall experimental design and the mice processed for mRNA sequencing at each time point (1 w, 1m, 3m, 6m and 9m). Red dot: mice treated with cigarette smoke; blue dots: mice treated with fresh air. The arrow below the timeline indicates the perturbed processes or pathways at each time point. (**c**) Heat map showing the level of immune cell infiltration of lung cells extracted from cigarette-treated mice across 5 time points. (**d**) Heat map showing the expression of immune-related genes during coal smoke treatment. Genes marked red were candidate therapeutic targets based on literatures^26-30^.


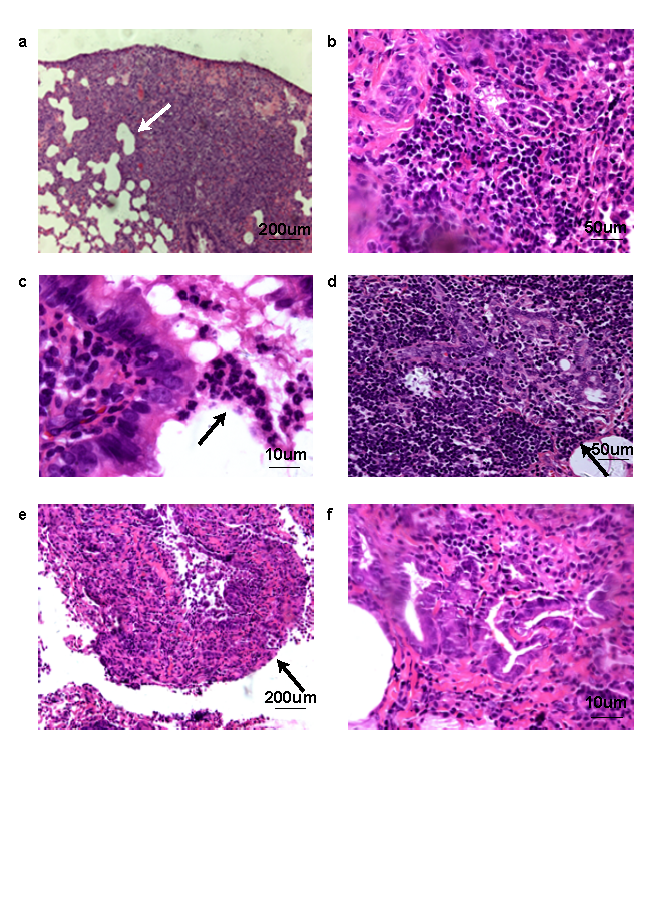


**Supplementary Fig. 7|** **Hematoxylin-eosin staining of pathological section of lung tissues from rats after coal smoke treatment.** (**a**) Pathological section of lung tissue from rats at 8 w (20x). Carbon was deposited in the lung tissue, the alveolar wall became thinner, and some alveolar walls were broken and merged into bullae. (**b**) Pathological section of lung tissue from rats at 8 w (100x). Some inflammatory cells were observed around the alveolar wall. (**c**) Pathological section of lung tissue from rats at 20 w (400x). Some carbon deposition and partial hetero-cell aggregation could be seen near the pleura. (**d**) Pathological section of lung tissue from rats at 24 w (100x). Some inflammatory cells infiltrated the alveolar wall. (**e**) Pathological section of lung tissue from rats at 20 w (20x). Inflammatory pseudotumour. (**f**) Pathological section of lung tissue from rats with 24-w (400x) inflammatory pseudotumours. Each representative result (a-f) was confirmed in 3 biological replicates (rats).


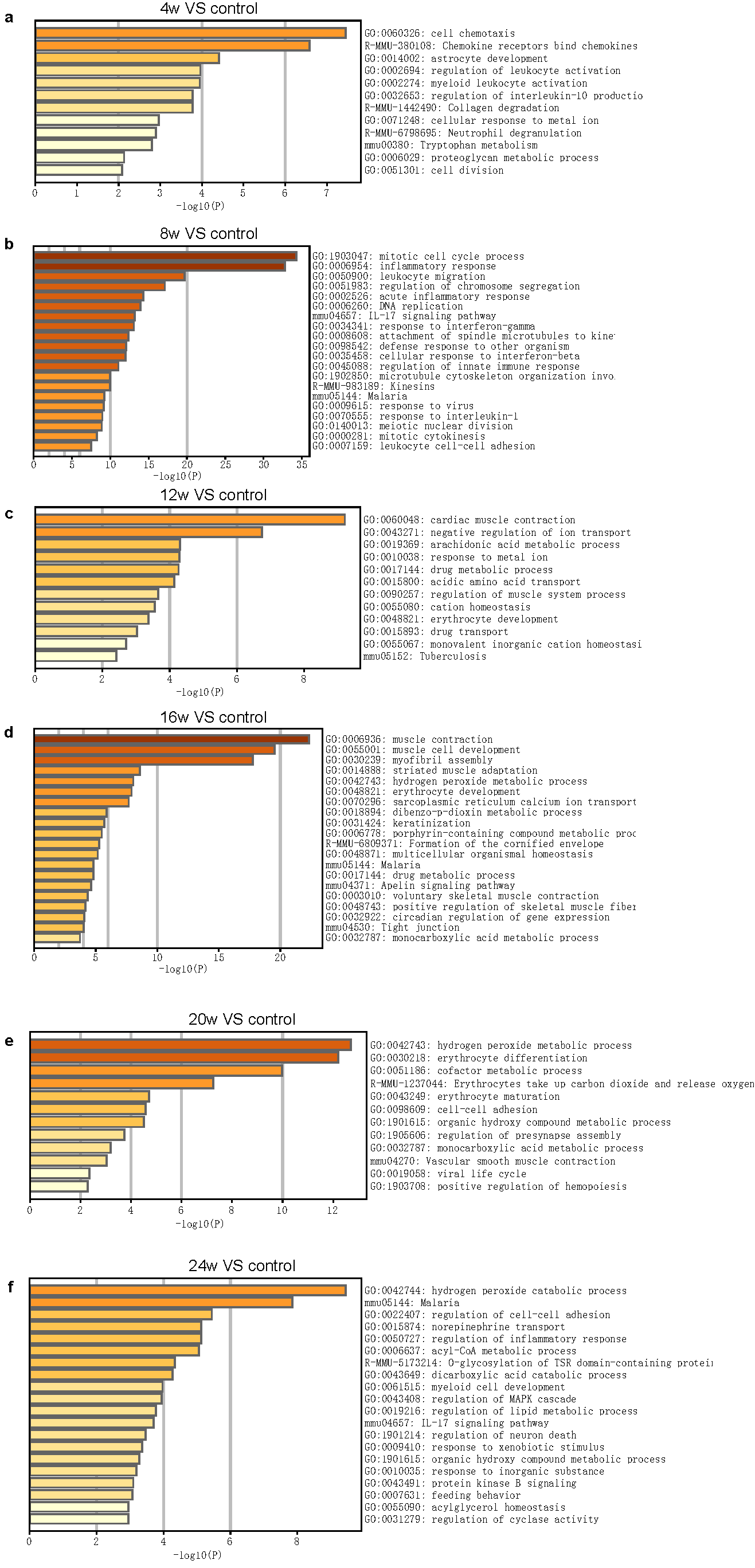


**Supplementary Fig. 8|** **Altered biological processes enriched in each time point in rat_coal model.** Colored bar plot showing the statistically enriched terms (GO/KEGG terms, canonical pathways) by comparing the transcriptomes of smoke-treated rats with those of 6 control rats from each time point 4w (a), 8 w (b), 12w (c), 16w (d), 20w (e) and 24w (f). Accumulative hypergeometric *P* values were adjusted with FDR. FDR < 0.05 was set to determine the significance.


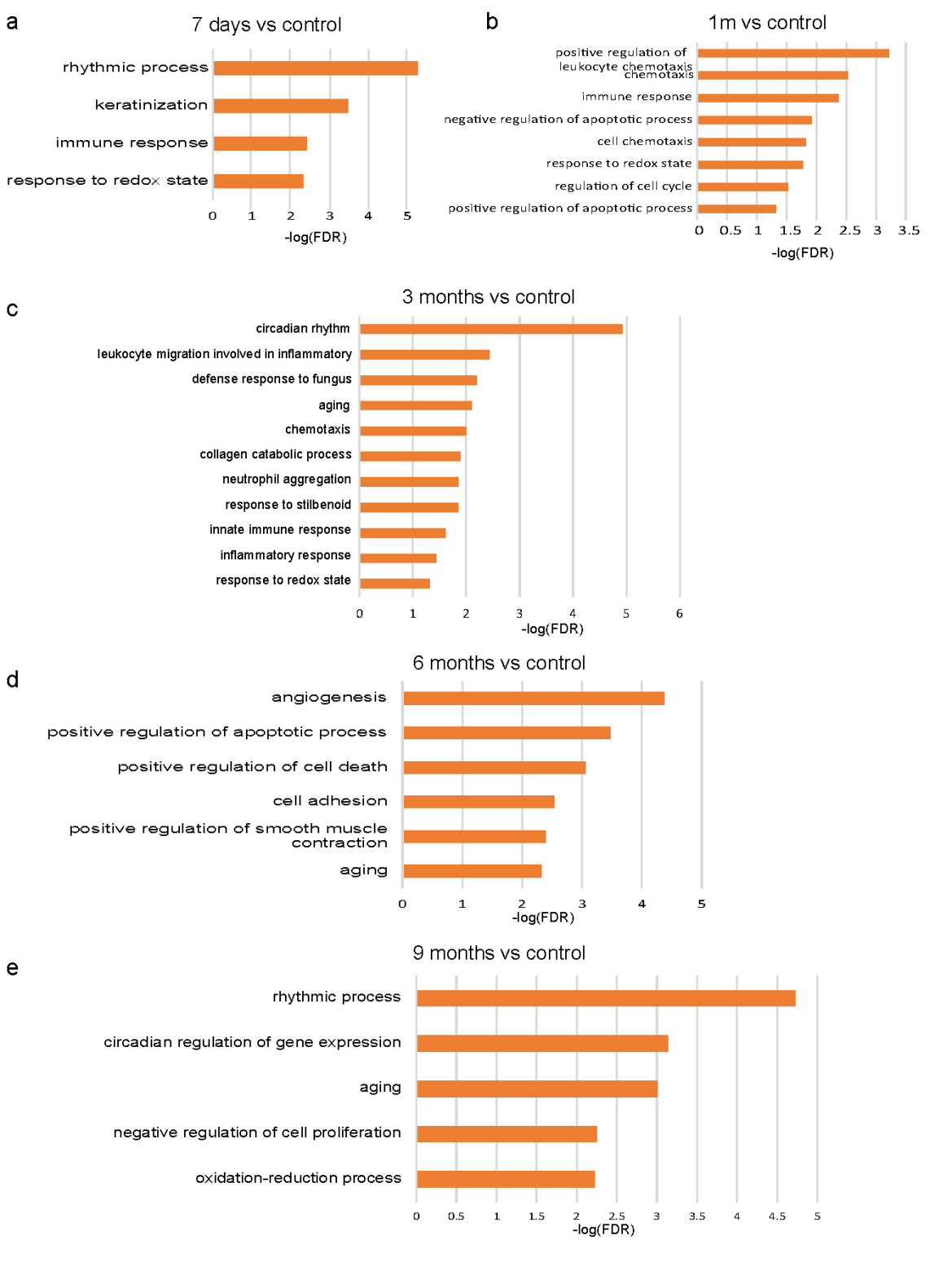


**Supplementary Fig. 9|** **Altered biological processes enriched in each time point in mouse_cigarette model.** Colored bar plot showing the statistically enriched GO terms by comparing the transcriptomes of cigarette-smoke-treated mice with control mice from each time point 1w (a), 1m (b), 3m (c), 6m (d) and 9m (e). Accumulative hypergeometric p-values were adjusted with FDR. FDR < 0.05 was set to determine the significance.

**References:**

1. Lawrence, M.S. *et al.* Discovery and saturation analysis of cancer genes across 21 tumour types. *Nature* **505**, 495-501 (2014).

2. Tamborero, D., Gonzalez-Perez, A. & Lopez-Bigas, N. OncodriveCLUST: exploiting the positional clustering of somatic mutations to identify cancer genes. *Bioinformatics* **29**, 2238-44 (2013).

3. Gaujoux, R. & Seoighe, C. A flexible R package for nonnegative matrix factorization. *BMC Bioinformatics* **11**, 367 (2010).

4. Mayakonda, A., Lin, D.C., Assenov, Y., Plass, C. & Koeffler, H.P. Maftools: efficient and comprehensive analysis of somatic variants in cancer. *Genome Res* **28**, 1747-1756 (2018).

5. Favero, F. *et al.* Sequenza: allele-specific copy number and mutation profiles from tumor sequencing data. *Ann Oncol* **26**, 64-70 (2015).

6. Mermel, C.H. *et al.* GISTIC2.0 facilitates sensitive and confident localization of the targets of focal somatic copy-number alteration in human cancers. *Genome Biol* **12**, R41 (2011).

7. Carter, S.L. *et al.* Absolute quantification of somatic DNA alterations in human cancer. *Nat Biotechnol* **30**, 413-21 (2012).

8. Hoshida, Y., Brunet, J.P., Tamayo, P., Golub, T.R. & Mesirov, J.P. Subclass mapping: identifying common subtypes in independent disease data sets. *PLoS One* **2**, e1195 (2007).

9. Hanzelmann, S., Castelo, R. & Guinney, J. GSVA: gene set variation analysis for microarray and RNA-seq data. *BMC Bioinformatics* **14**, 7 (2013).

10. Li, H. & Durbin, R. Fast and accurate long-read alignment with Burrows-Wheeler transform. *Bioinformatics* **26**, 589-595 (2010).

11. Li, H. A statistical framework for SNP calling, mutation discovery, association mapping and population genetical parameter estimation from sequencing data. *Bioinformatics* **27**, 2987-2993 (2011).

12. McKenna, A. *et al.* The Genome Analysis Toolkit: A MapReduce framework for analyzing next-generation DNA sequencing data. *Genome Research* **20**, 1297-1303 (2010).

13. Cibulskis, K. *et al.* Sensitive detection of somatic point mutations in impure and heterogeneous cancer samples. *Nature Biotechnology* **31**, 213-219 (2013).

14. Lek, M. *et al.* Analysis of protein-coding genetic variation in 60,706 humans. *Nature* **536**, 285-+ (2016).

15. Wang, K., Li, M. & Hakonarson, H. ANNOVAR: functional annotation of genetic variants from high-throughput sequencing data. *Nucleic Acids Res* **38**, e164 (2010).

16. Schenck, R.O., Lakatos, E., Gatenbee, C., Graham, T.A. & Anderson, A.R.A. NeoPredPipe: high-throughput neoantigen prediction and recognition potential pipeline. *BMC Bioinformatics* **20**, 264 (2019).

17. Shukla, S.A. *et al.* Comprehensive analysis of cancer-associated somatic mutations in class I HLA genes. *Nat Biotechnol* **33**, 1152-8 (2015).

18. Jurtz, V. *et al.* NetMHCpan-4.0: Improved Peptide-MHC Class I Interaction Predictions Integrating Eluted Ligand and Peptide Binding Affinity Data. *J Immunol* **199**, 3360-3368 (2017).

19. Alexandrov, L.B. *et al.* The repertoire of mutational signatures in human cancer. *Nature* **578**, 94-101 (2020).

20. Roth, A. *et al.* PyClone: statistical inference of clonal population structure in cancer. *Nat Methods* **11**, 396-8 (2014).

21. Huang, D.W., Sherman, B.T. & Lempicki, R.A. Systematic and integrative analysis of large gene lists using DAVID bioinformatics resources. *Nature Protocols* **4**, 44-57 (2009).

22. Zhou, Y. *et al.* Metascape provides a biologist-oriented resource for the analysis of systems-level datasets. *Nat Commun* **10**, 1523 (2019).

23. Robinson, M.D., McCarthy, D.J. & Smyth, G.K. edgeR: a Bioconductor package for differential expression analysis of digital gene expression data. *Bioinformatics* **26**, 139-140 (2010).

24. Charoentong, P. *et al.* Pan-cancer Immunogenomic Analyses Reveal Genotype-Immunophenotype Relationships and Predictors of Response to Checkpoint Blockade. *Cell Rep* **18**, 248-262 (2017).

25. Yang, J. *et al.* Research and Design of an Experimental Apparatus Based on the "Open Fireplace" in Xuanwei District. *Journal of Biomedical Engineering* **33**, 101-107 (2016).

26. Ning, Z., Liu, K. & Xiong, H. Roles of BTLA in Immunity and Immune Disorders. *Front Immunol* **12**, 654960 (2021).

27. Holmes, K., Roberts, O.L., Thomas, A.M. & Cross, M.J. Vascular endothelial growth factor receptor-2: structure, function, intracellular signalling and therapeutic inhibition. *Cell Signal* **19**, 2003-12 (2007).

28. Mittal, D. *et al.* CD96 Is an Immune Checkpoint That Regulates CD8(+) T-cell Antitumor Function. *Cancer Immunol Res* **7**, 559-571 (2019).

29. Fong, L. *et al.* Adenosine 2A Receptor Blockade as an Immunotherapy for Treatment-Refractory Renal Cell Cancer. *Cancer Discov* **10**, 40-53 (2020).

30. Zhang, J., Yan, W., Duan, W., Wuthrich, K. & Cheng, J. Tumor Immunotherapy Using A2A Adenosine Receptor Antagonists. *Pharmaceuticals (Basel)* **13**(2020).
